# Supplementary material for: Quantification of Enterocytozoon hepatopenaei (EHP) in Penaeid Shrimps from Southeast Asia and Latin America Using TaqMan Probe-Based Quantitative PCR
Source: Pathogens. 2019 Nov 12;8(4):233. doi: 10.3390/pathogens8040233 (PMC6963587; doi:10.3390/pathogens8040233)

Supplementary Fig. 1.

Standard curve of *Enterocytozoon hepatopenaei* (EHP) qPCR, based on a standard curve of the copy number of β-tubulin gene versus the qPCR Ct (threshold cycle). Purified pEHP-tubB-2 plasmid was serially diluted from 10^8 to 10^2 copies per reaction and used as a template in the qPCR. The resulting Ct values are plotted against the logarithm of their respective copy number.

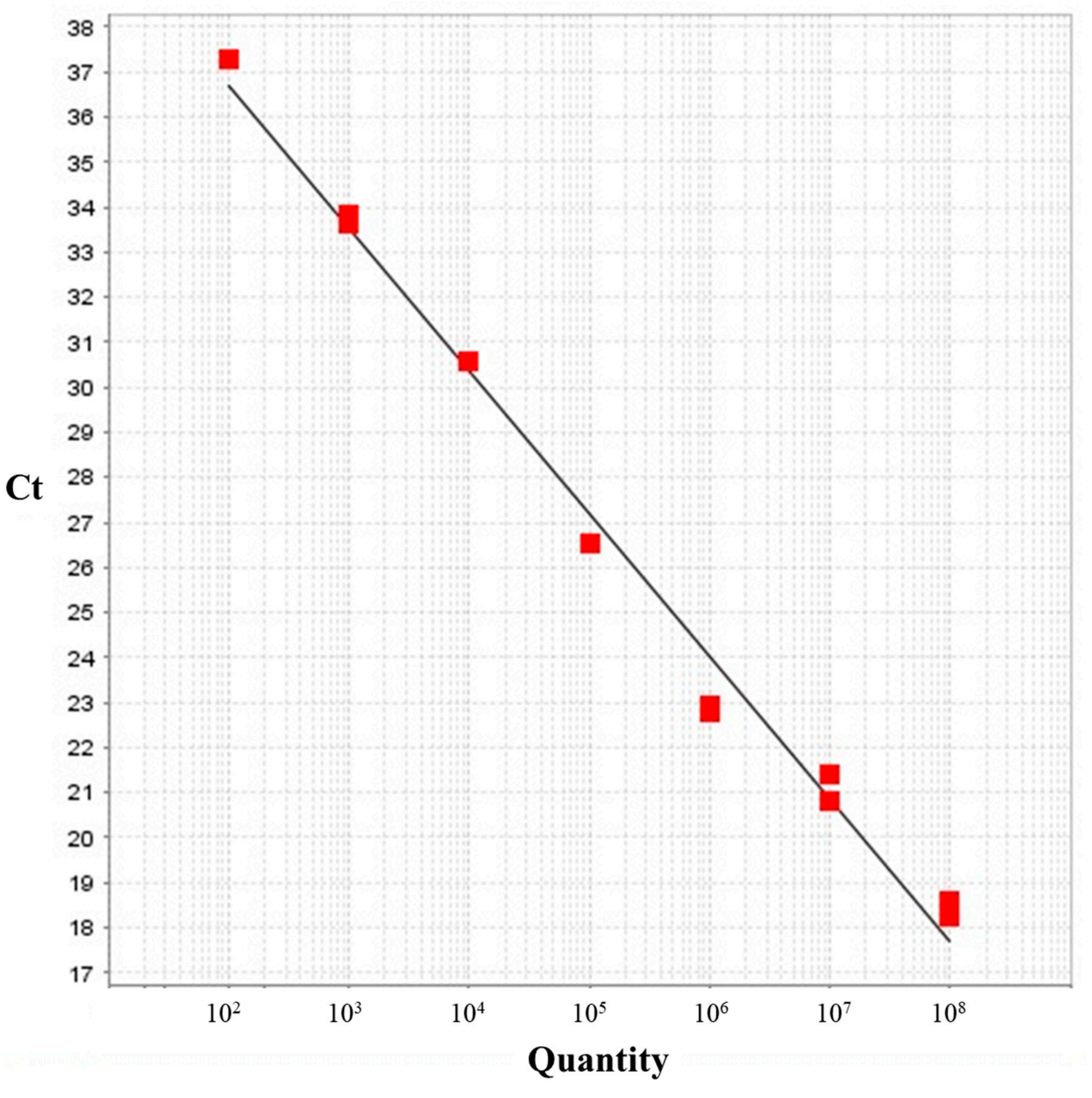

Supplementary Fig. 2.

Amplification plots of *Enterocytozoon hepatopenaei* (EHP) samples were detected by qPCR (with standards). DNAs were prepared from hepatopancreas (HP), feces, artemia, and water.

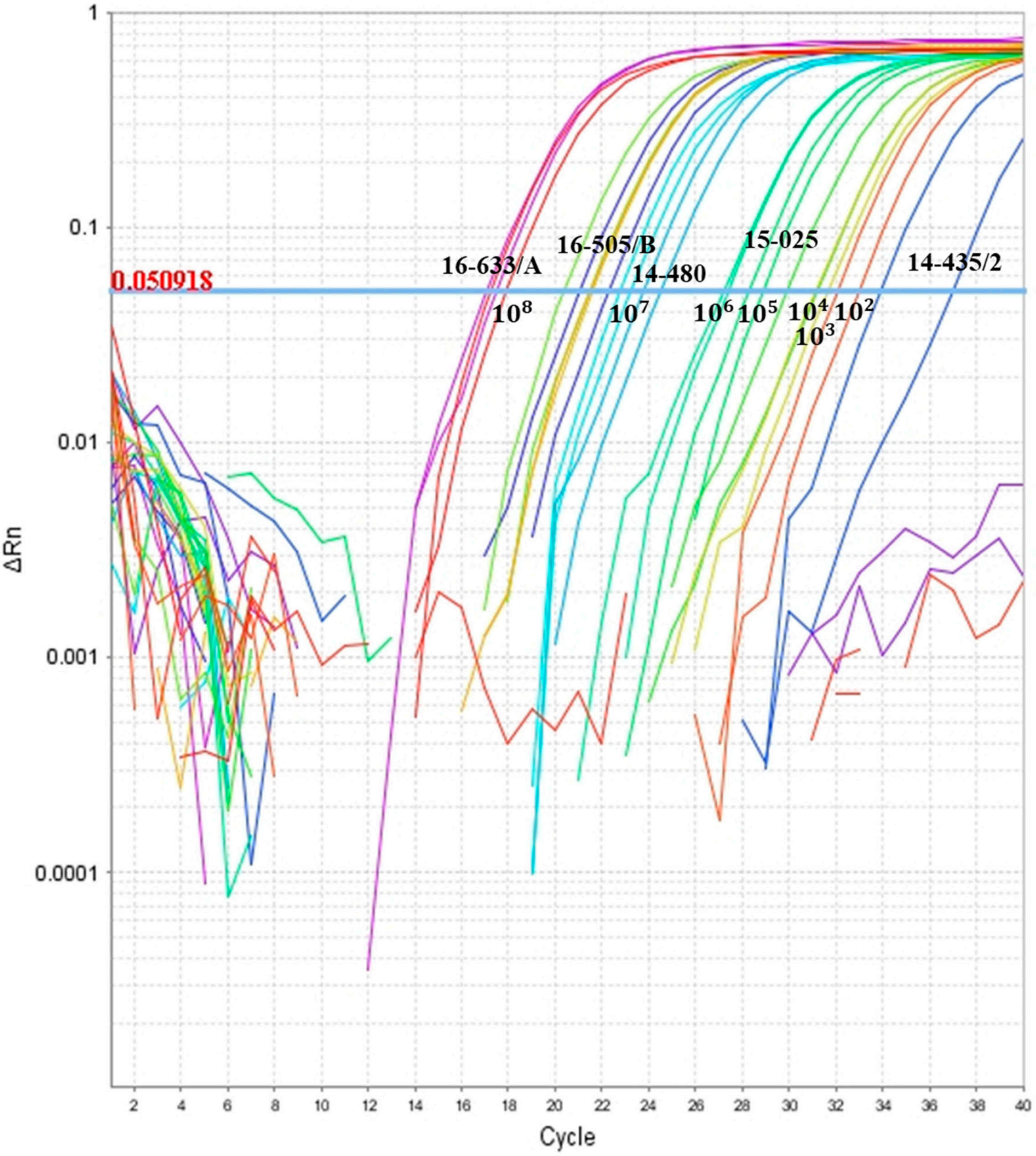

Supplement: Supplementary file 1 [file pathogens-08-00233-s001.pdf]
